# A systematic approach to 3D echocardiographic assessment of the aortic root

**DOI:** 10.21542/gcsp.2018.12

**Published:** 2018-06-30

**Authors:** Andreas Hagendorff, Stephan Stoebe, Bhupendar Tayal

**Affiliations:** 1 Dep. of Cardiology, University Hospital Leipzig, Germany; 2 Dep. of Cardiology, Aalborg University Hospital, Denmark

## Introduction

Severe aortic regurgitation (AR) and/or severe abnormalities of the aortic root and the tubular ascending aorta (TAA) are indications for surgical treatment^1–9^. The correct diagnosis, the choice of optimal treatment, as well as optimal timing of surgery, mainly depend on findings obtained by echocardiography - which is usually the initial diagnostic modality applied in clinical practice^10–21^. Therefore, an appropriate morphological and functional quantification of the aortic valve (AV) and the aortic root complex is required. Aside from the need of standardization to provide a precise objective evaluation, the use of modern echocardiographic technologies - especially 3D-echocardiography - are less often implemented in clinical routine. The present manuscript focuses on the advantages of transthoracic and transesophageal 3D-echocardiography (TTE, TEE) for an improved assessment of the AV and the aortic root complex to provide accurate and comprehensive measurements for making the correct diagnosis and defining further therapeutic strategies.

## 1. Quantification of AR

### 1.1. Applying conventional 2D-echocardiography

AR is qualitatively diagnosed by suspicious morphological findings of the cusps, by diastolic regurgitant jet formation into the left ventricle (LV) using color-coded Doppler echocardiography, by retrograde signals of transvalvular velocities using continuous-wave (CW) Doppler echocardiography, or by diastolic reversal flow of the arterial velocities determined in the proximal descending aorta or in the left subclavian artery using pulsed-wave (PW) Doppler echocardiography^11,21–23^.

All qualitative parameters simply document the presence of AR without providing any information about severity. In particular, the size and the shape of the regurgitant jet area are generally not recommended for quantification of AR severity, mainly due to considerable effects of methodological issues, ultrasound system settings, and individual hemodynamic situations on the color flow signal^7,16–18,21^.

According to the current guidelines several semi-quantitative parameters are recommended for quantification of AR severity^7,16–18,21,24,25^. An overview of generally recommended semi-quantitative parameters including their methodological limitations is given in Table 1. The grading of AR severity by several semi-quantitative parameters is highly debatable, because all these parameters can be extremely misleading under certain conditions.

**Table 1:** Limitations of quantification of aortic regurgitation using conventional 2D echocardiography.

Parameter	Technique	Limitations
Vena contracta	Narrowest jet width distal to valve preferably in the parasternal long-axis view	• Jet origin with respect to commissures • Multiple jets
D_jet_/D_LV OT_	Ratio between the regurgitant jet diameter and the LVOT diameter preferably in parasternal long-axis view	• Jet origin with respect to commissures
A_jet_/A_LV OT_	Ratio between the regurgitant jet area and the LVOT area in the parasternal short-axis view	• Eccentric jet formations
Proximal flow convergence and effective regurgitant orifice area (EROA) by PISA	Assessment of the EROA and the diameter of the flow convergence zone and regurgitant flow velocities preferably in the apical long axis view	• Non-hemispheric shape of the jet • Multiple jets • Eccentric jet formations
Pressure-half-time	Pressure fall of the trans-valvular flow preferably in the apical long axis view	• Doppler angulation • Depends on LV filling pressure
Maximum diastolic flow reversal and ratio of the velocity time integrals in systole/diastole (VTI_sys_/VTI_dia_) in the proximal descending aorta or the left subclavian artery	Using pulse Doppler technique in the proximal descending aorta	• Doppler angulation • Alterations due to aortic compliance

With respect to the limitations of the semi-quantitative approach, the diagnosis of severe AR cannot only be made semi-quantitatively, or by only one semi-quantitative parameter. The assessment of the effective regurgitant orifice area (EROA) and the regurgitant volume (RVol) by the PISA method seems to be preferred by the current guidelines, but is rarely applicable due to methodological limitations^16–18,21^. Reliable results by this approach can only be assumed in the presence of a prolapse of the right coronary cusp (RCC) if Doppler angulation of the jet formation is optimal using the left parasternal acoustic window - otherwise EROA, RVol will generally be overestimated. In general, eccentric jets and jets with a ‘coanda effect’ should be analyzed carefully to avoid over- or underestimation of AR severity.

It is well known that left and right ventricular volume (LV, RV) analyses are necessary for grading of severity in patients with presumably relevant valvular heart disease. In particular, patients with AR LV diameters and LV volumes have to be assessed quantitatively^16–19,21,26,27^. In these AR patients, accurate volume analyses are necessary for the determination of the total (SV_t_) and effective stroke volume (SV_e_), the RVol (SV_t_–SV_e_), and the regurgitant fraction (RF).

The RF is the primary and most important parameter of the quantitative approach, and is calculated by the following equation:

\begin{eqnarray*}\mathrm{RF}[\text{%}]=({\mathrm{SV }}_{\mathrm{t}}-{\mathrm{SV }}_{\mathrm{e}})/{\mathrm{SV }}_{\mathrm{t}}\times 100. \end{eqnarray*}

**Figure 1.**
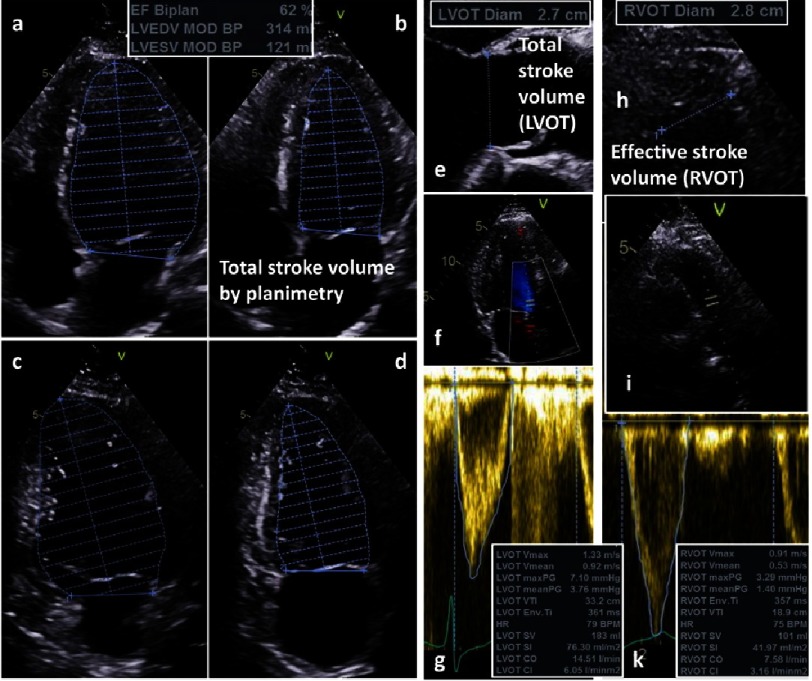
Conventional assessment of total stroke volume (SV_t_) and effective stroke volume (SV_e_) by 2D-echocardiography; determination of SV_t_ by LV planimetry using the modified Simpsons’ rule (4-chamber view: a - diastole; b - systole; 2-Chamber view: c - diastole, d -systole) and by Doppler echocardiography (e - LVOT diameter; f - position of the sample volume; g - pulsed-wave Doppler spectrum and VTI of LVOT flow velocities); determination of SV_e_ by Doppler echocardiography (h - RVOT diameter; i - position of the sample volume; k - pulsed-wave Doppler spectrum and VTI of RVOT flow velocities).

The calculation of the individual RF is the only possible objective approach to assess the AR severity with respect to the actual hemodynamic state of the patient. However, the accurate quantitative assessment of the RF is very difficult using conventional 2D-echocardiography because basic methodological aspects have to be considered, e.g., standardized sectional planes to correctly perform LV planimetry, accurate sectional planes of the left and right ventricular outflow tract (LVOT, RVOT) to correctly determine the diameters, as well as to correctly position the sample volume in the respective locations at the correct time points of the cardiac cycle (Figure 1).

SV_t_ can be estimated by LV planimetry using the modified Simpson’s rule or by determination of the LVOT diameter (D_LV OT_) and the velocity time integral of the LVOT during the ejection period (VTI_LV OT_) using PW Doppler echocardiography (SV_LV OT_ = 0.785 × D_LV OT_^2^ × V TI_LV OT_).

SV_e_ can be assessed by determination of the RVOT diameter (D_RV OT_) and the VTI_RV OT_ during the ejection period using PW Doppler echocardiography (SV_RV OT_ = 0.785 × D_RV OT_^2^ × V TI_RV OT_).

**Figure 2.**
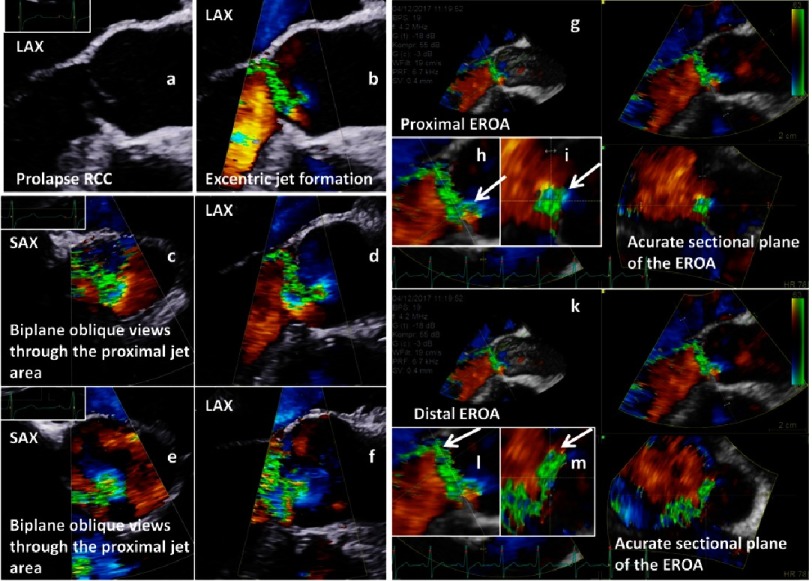
Assessment of the EROA by 2D-echocardiography (a-f) and 3D-echocardiography (g-m); simultaneous long axis view (LAX) of an AV prolapse during diastole (a - native; b - color-coded); color-coded biplane views of the regurgitant jet documenting oblique sectional planes of the regurgitant area in the short axis view (SAX) (c - SAX; d - LAX); second biplane color-coded approach to document the regurgitant jet area (e - SAX; f - LAX); 3D analysis of the entry of the regurgitant jet by post-processing of 3D data sets (g), longitudinal documentation of the central jet formation (h) and accurate perpendicular sectional plane for determination of proximal EROA (i); 3D analysis of the exit of the regurgitant jet by post-processing of 3D data set (k), longitudinal documentation of the central jet formation (l) and accurate perpendicular sectional plane for determination of distal EROA (m).

### 1.2. Advantages of applying 3D-echocardiography

Potentially the best semi-quantitative approach of grading AR severity is the accurate planimetry of the EROA, which corresponds to the 2D color-coded regurgitant orifice area perpendicular to the jet stream characterized by vena contracta using 2D-echocardiography^28–33^. However, the EROA can usually not be visualized with accuracy in conventional 2D sectional planes because the proximal jet direction and the EROA depend on AV morphology, and usually oblique sectional planes through the regurgitant jets are documented by 2D-echocardiography. Thus, the accurate alignment of the EROA can only be performed in the most appropriate sectional plane at the most appropriate point in the cardiac cycle by post-processing using 3D-echocardiography (Figure 2).

**Figure 3.**
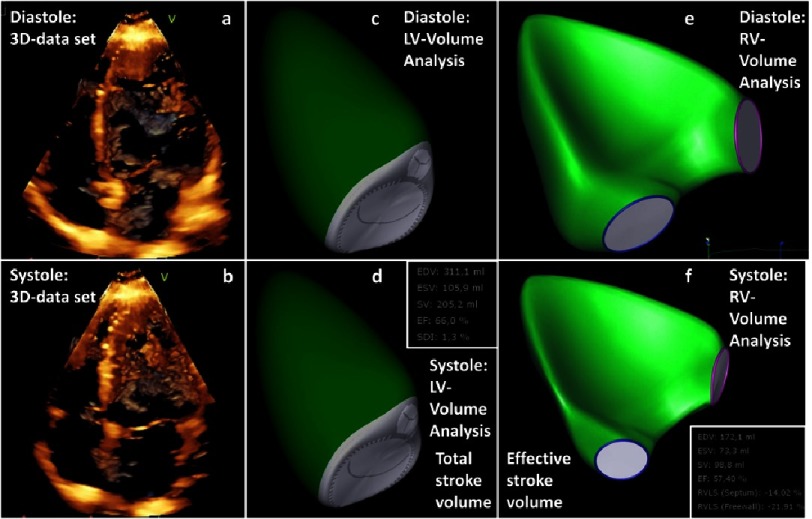
Determination of regurgitant fraction (RF) by 3D-echocardiography. 3D 4-chamber view during diastole (a) and systole (b); determination of LV volumes and total stroke volume (SV _t_) by post-processing of 3D data set during diastole (c) and systole (d); determination of RV volumes and effective stroke volume (SV_e_) by post-processing of 3D-data set during diastole (e) and systole (f); RF is calculated by RF[%] = [(SV _t_ − SV _e_)∕SV _t_] × 100.

Further, LV/RV volumes can be assessed by 3D transthoracic echocardiography to overcome the limitations of 2D volume analyses and to enable a more accurate estimation of SV_t_ and SV_e_ using high quality 3D data sets (Figure 3)^34–40^. However, accurate delineation of the endocardial contour is a prerequisite for these measurements, and training in several methodological issues is required prior to starting 3D-echocardiography in patients with valvular heart diseases, e.g., the image optimization to visualize sharp contour delineation, the correct endocardial contour delineation including the trabecula into the LV cavity, and the correct labeling of the mitral and aortic annulus to assess the LV base.

The 3D assessment of RV stroke volume (SV_e_) and LV stroke volume (SV_t_) enables an objective calculation of RF. The quantitative assesment of RF by 3D-echocardiography shows good agreement to RF assessment using cardiac magnetic resonance imaging (cMRI)^41–46^.

## 2. Assessment of morphology of aortic valve and aortic root

### 2.1. Applying conventional 2D-echocardiography

**Figure 4.**
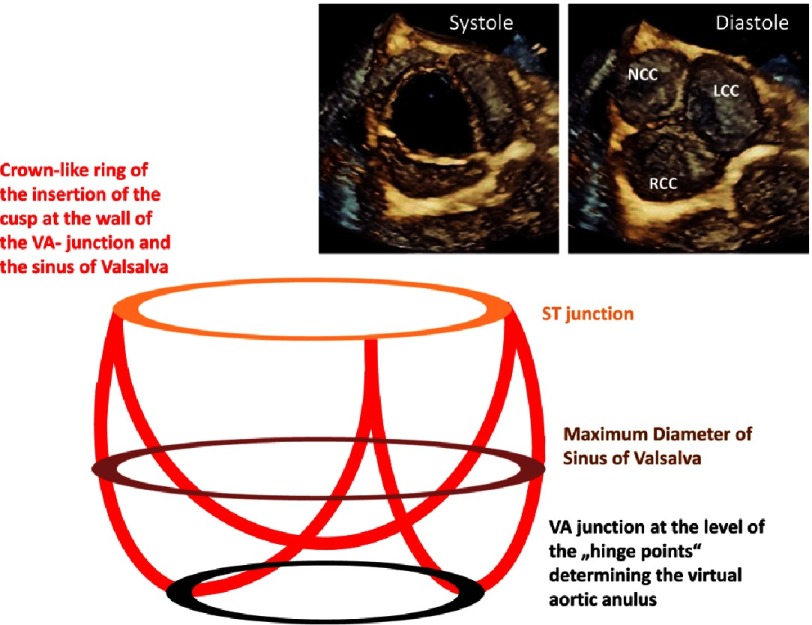
Simplified scheme of the aortic root showing the level of the sinotubular (ST) junction, the maximum diameter of the sinuses of Valsalva, and the ventriculo-arterial (VA) junction at the hinge points of the cusps. The crown-like ring of the cusps insertion with the aortic root is depicted. En-face views of the AV are shown during systole and diastole.

The main difficulty in assessment of the aortic root morphology by 2D-echocardiography is the accurate alignment of the sectional plane through the respective cardiac structures^47^. Thus, the AV and the aortic root is a complex multi-dimensional entity which can be better analyzed with 3D-echocardiography (Figure 4)^48–54^.

**Figure 5.**
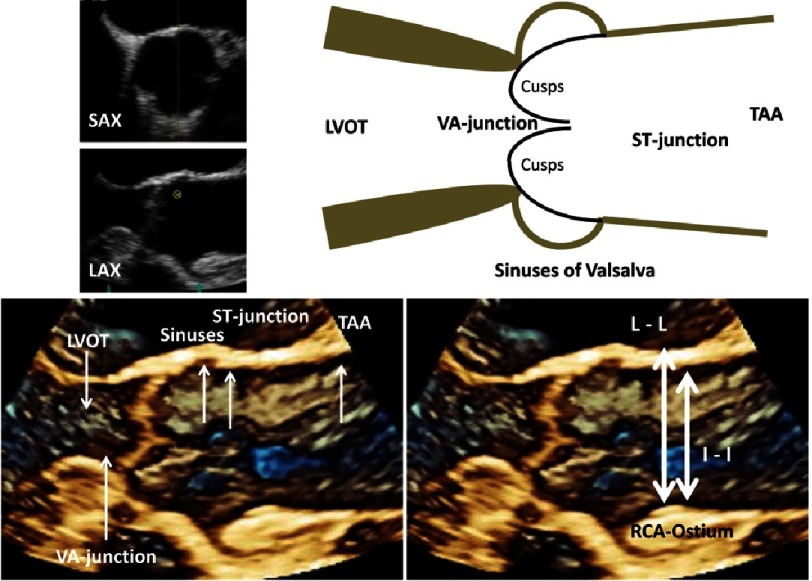
Scheme of the LVOT and the aortic root including 3D TEE-Illustrations of the AV and the aortic root during diastole (left below). The characteristic structures are labeled by white arrows (abbreviations see text). The differences between leading edge to leading edge (L-L) and inner edge to inner edge (I-I) are documented for the diameter of ST-junction (right below).

The assessment of the aortic root complex should exactly provide the maximum diameter and size of the LVOT, aortic annulus, sinuses of Valsalva, sinutubular junction (ST) and ascending aorta. All these measurements should be performed according to the recent recommendations using the leading edge to leading edge (L-L) method during end-diastole (Figure 5)^10,55,56^.

With respect to the equal or similar spatial resolution of echocardiography in comparison to magnetic resonance and computed tomography, it should be discussed whether or not diameter measurements by 3D-echocardiography should be performed using the inner edge to inner edge (I-I) method, which is applied using magnetic resonance and computed tomography^21,57,58^. If the correct sectional planes are provided using 2D- or 3D-echocardiography, the diameter measurements should be performed by the same method. However, diameters determined by 2D-echocardiography have been shown to be underestimated in clinical routine in comparison to cMRI or computed tomography, which is obviously due to foreshortening in 2D-echocardiography^21,29,34,35,40,46,50,55,56,59,60^.

Further, it is important at which time point of the cardiac cycle these measurements are performed. Despite the recommendations to perform these measurements during diastole, there is a rationale to assess the maximum diameters during systole, when all these structures will have their largest dimensions. The diameters of the LVOT, the basal aortic annulus and the aortic root are larger during systole because of the stretching of these structures at maximum blood pressure in comparison to diastole at low blood pressure, especially in patients with relevant AR and in younger patients in whom the ventricular-aortic junction is formed by non-calcified tissue^51,59,61–65^.

**Figure 6.**
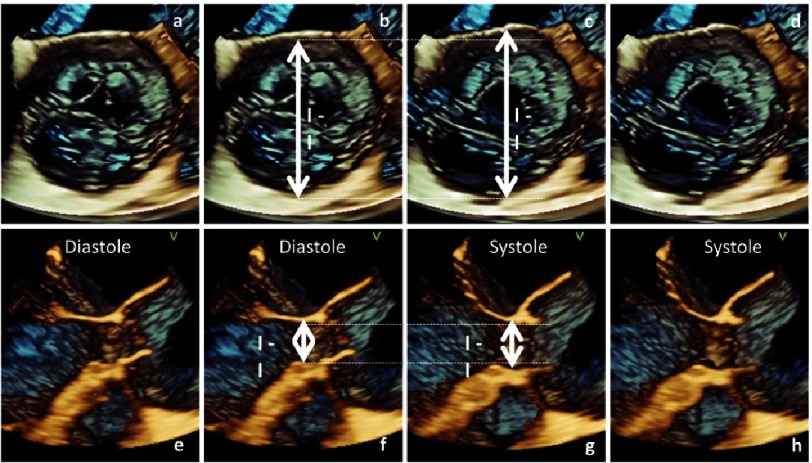
Documentation of the changes of the ST-junction (a-d) and VA-junction (e-h) diameter in a patient with an aneurysm of the ascending aorta and severe aortic regurgitation. En-face view of the AV from the level of the ST-junction is shown at end-diastole (a) including I-I diameter (b); En-face view of the AV from the level of the ST-junction is shown at mid-systole (d) including I-I diameter (c); the difference of the ST-junction diameter is illustrated by dotted lines. Longitudinal view of the AV valve and VA-junction is shown at end-diastole (e) including I-I diameter (f); Longitudinal view of the AV and VA-junction is shown at mid-systole (h) including I-I diameter (g); the difference of the VA-junction diameter is illustrated by dotted lines.

In healthy subjects it is known that there is a significant difference of more than 2 mm between the diameter at systole and diastole^45,66^. It can be assumed that these differences might be more pronounced in AR patients due to volume overload in the presence of high regurgitant volumes. With respect to surgery there is a pathophysiological rationale that diameter measurements of the AV and the aortic root complex should be performed at mid-systole, although this has to be proven by further studies (Figure 6).

**Figure 7.**
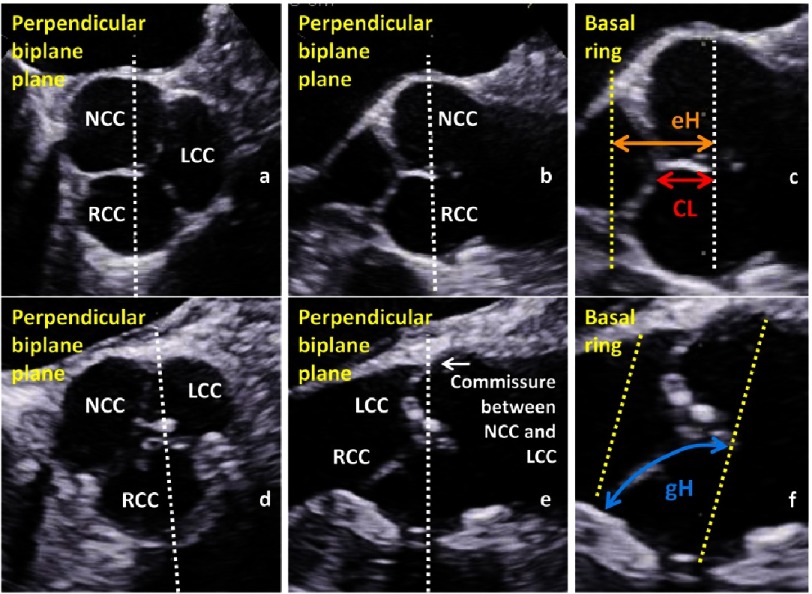
Short axis view with labeling of the non-(NCC), left-(LCC) and right coronary cusp (RCC) (a) and perpendicular long axis view (b) of a normal AV, documentation and labeling of the coaptation length (CL) and effective height (eH) in the long axis view (c); short axis view (d) and perpendicular long axis view (e) of a mild calcified AV, documentation and labeling of the geometric height (gH) in the long axis view of the RCC (f).

The assessment of the geometry and configuration of the cusps should provide the coaptation length (CL) and the effective and geometric height (eH, gH) which have to be determined during diastole (Figure 7)^67–71^. The CL describes the distance of the adjacent cusps when the AV is closed (CL normal values: >2–5 mm). The eH describes the difference between the annular plane and the free margin of each cusp (eH normal values: >11 mm). The gH describes the distance of the curved length of each cusp during diastole from the nadir of the sinus to the central part of the free margin of each cusp (gH normal values might be >20 mm, but is not presented in the literature so far).

The AV and the aortic root complex are usually symmetric, but the standardized longitudinal sectional plane by 2D-echocardiography does rarely correspond to the central position of the longitudinal sectional plane through this sophisticated symmetrical structure. According to anatomical aspects the caudal part of the aortic root complex is formed by the nadirs or the hinge points of the cusps. The cranial part is limited by the level of the ST-junction which is normally characterized by the tips of the commissures between the cusps. The three cusps of a normal AV are attached to the aortic root in a crown-like curved fashion (Figure 4). Thus, the caudal border can be labeled as the basal ring which forms the ventricular-aortic junction, the sinuses of Valsalvae (non-coronary, left and right coronary sinus) are cranially limited by the ST-junction, which is followed by the proximal part of the tubular ascending aorta (TAA)^48,51,53,54^.

The so-called ‘virtual basal ring’ represents a circular or elliptic shaped line connecting the nadirs of the cusp insertion lines which are usually at the same level. The dimension of the basal ring varies between systole and diastole, depending on its extensibility^9,19,61–63,74^.

Depending on the sectional longitudinal planes through the aortic root complex, different levels of attachment between ventricular myocardium or the aortic wall and the cusps are shown. Thus, with respect to the orientation of the cusps, the central longitudinal sectional plane of the aortic root complex using 2D-echocardiography will present the nadir of the right coronary cusp (RCC) and the tip of the commissure between the left and non-coronary cusp (LCC, NCC), which leads to uncertainty in delineating the correct level of the basal aortic annulus.

The accurate sectional plane of the virtual annulus is perpendicular to the level of all hinge points. Thus, the general problem of 2D-echocardiography is *per se* the documentation of the central position of the longitudinal sectional plane through the aortic root complex, which should be perpendicular to the levels of all hinge points of the cusps, to be able to determine the largest diameters instead of diameters within secants.

**Figure 8.**
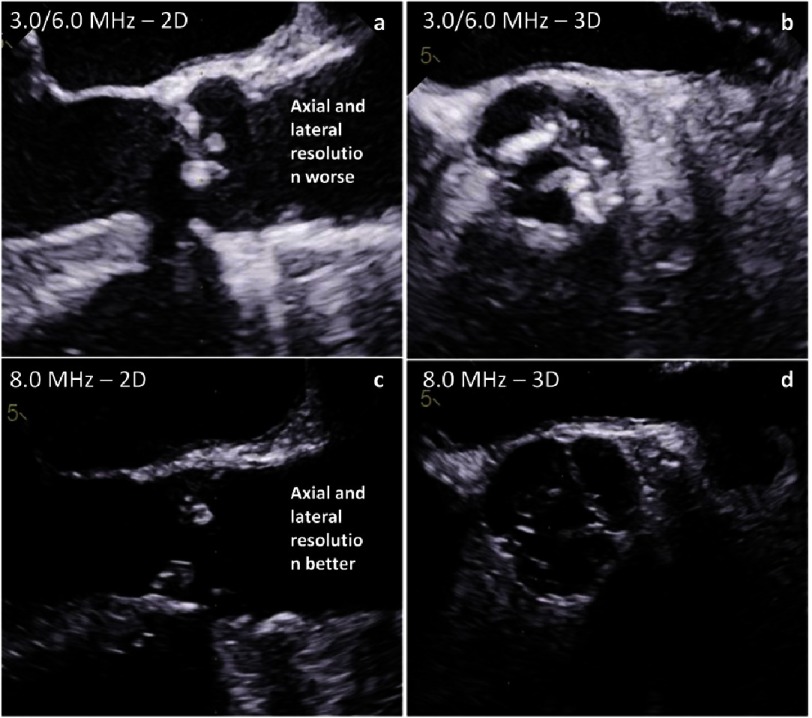
Differences of spatial resolution depending on frequency settings - long axis views (a, c) and short axis views (b, d) using low frequencies (harmonic 3.0/6.0 MHz - a, b) and high frequencies (8.0 MHz - c, d).

**Figure 9.**
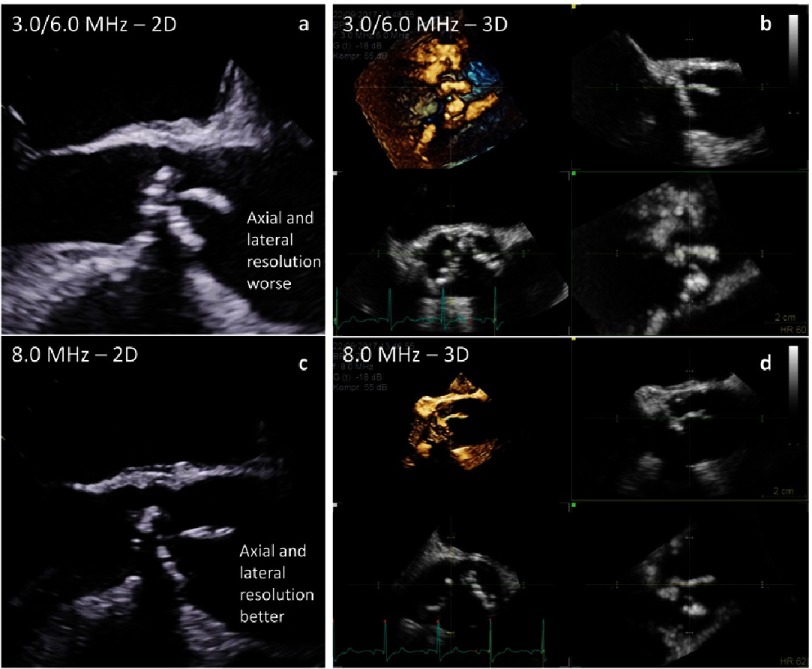
Differences of spatial resolution depending on frequency settings - long axis views (a, c) and 3D data sets (b, d) using low frequencies (harmonic 3.0/6.0 MHz - a, b) and high frequencies (8.0 MHz - c, d) in a patient with endocarditis.

The uncertainty of measuring the truly maximum diameter by 2D-echocardiography leads to the recommendation to use the L-L protocol instead of the I-I protocol. Thus, underestimation due to secants measurements will be reduced. However, this compromise should not be accepted because 2D-echocardiography has a better spatial resolution in comparison to other imaging modalities if correct sectional planes could be visualized by 2D-echocardiography. For better delineation of the contours high ultrasound frequencies should be used to improve spatial resolution despite reduction of penetration (Figures 8 and 9).

**Figure 10.**
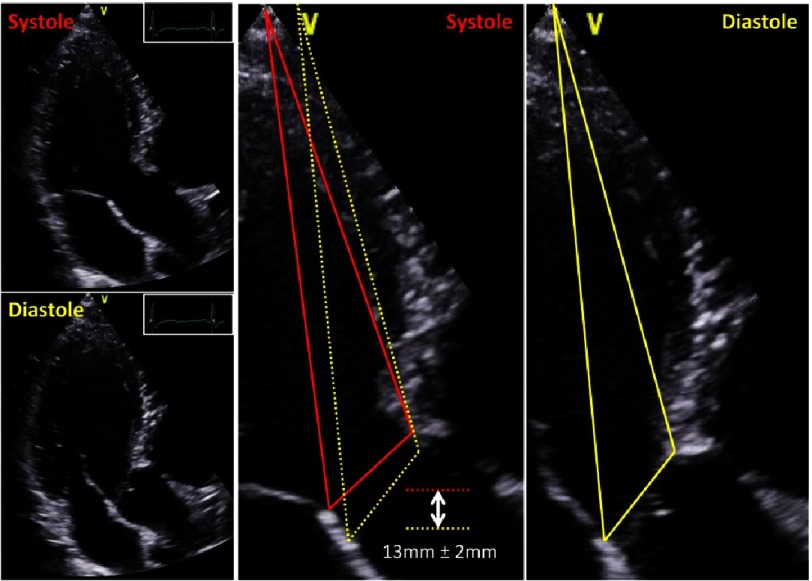
Caudal-cranial aortic annulus excursion between systole and diastole. Due to LV filling during diastole the distance between apex and AV-junction is more cranial in comparison to end-systole. Changes of aortic annulus position are illustrated by yellow lines during diastole and red lines during systole.

Dimensions of the aortic root complex should also be measured with respect to the dynamics of cardiac contraction, which cannot be corrected by 2D-echocardiography. Rotational and translational movements have to be considered for reliable measurements of the aortic root. The caudal-cranial aortic annulus excursion during the cardiac cycle is about 13 mm±2 mm to contribute an efficient cardiac output (Figure 10).

**Figure 11.**
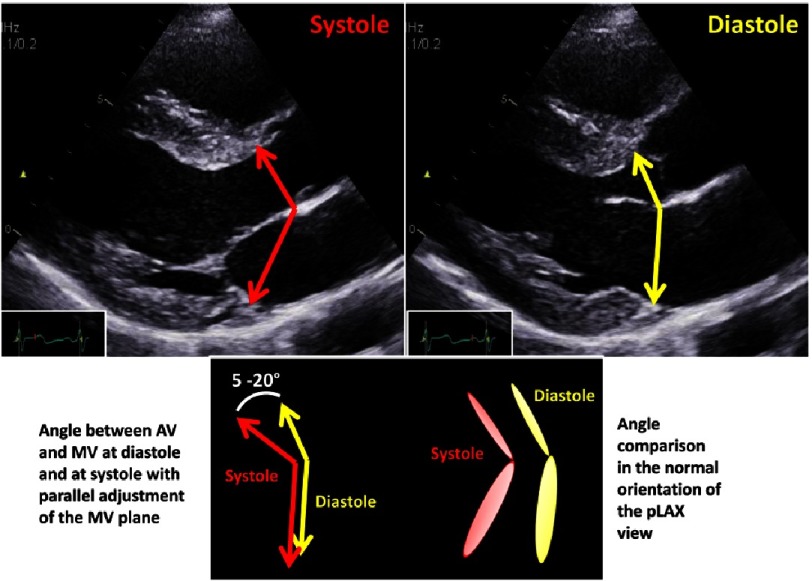
Angle differences between mitral and aortic annulus during systole (red) and diastole (yellow) including a scheme showing the angle difference of the mitral valve during systole and diastole (left below) with parallel adjustment in comparison to normal orientation of the annulus planes (right below).

In addition, the angle which is formed by the mitral and aortic annulus planes, is altered about 10°between systole and diastole providing normal LV contraction and relaxation dynamics (Figure 11). Thus, the measurements of the aortic root complex should be performed with respect to dynamics of cardiac contraction. Alignment of the diameters of LVOT, basal aortic ring, aortic root and TAA should be performed with respect to measurements performed at diastole or systole.

The longitudinal echocardiographic visualization of the normal tricuspid AV during diastole displays the CL between the RCC and the LCC or NCC. In addition, further parameters, which characterize cusp geometry, e.g., eH and gH, can only be determined for the RCC, because the standardized longitudinal sectional plane through the aortic root usually displays the central part of the RCC with the nadir of RCC (Figure 7).

In 2D-echocardiography the short axis view of the AV usually displays the number of cusps, the orientation of the commissures and the symmetry or asymmetry of the aortic root. However, the pitfall of 2D-echocardiography is to present a sectional short axis view which is exactly perpendicular to the central longitudinal axis of the aortic root or parallel to the virtual basal aortic annulus. The normal size of the aortic root is characterized by a diameter of the ST-junction, which is 15–20% ≤ to the diameter of the virtual aortic annulus^49^.

### 2.2. Advantages applying 3D-echocardiography

**Figure 12.**
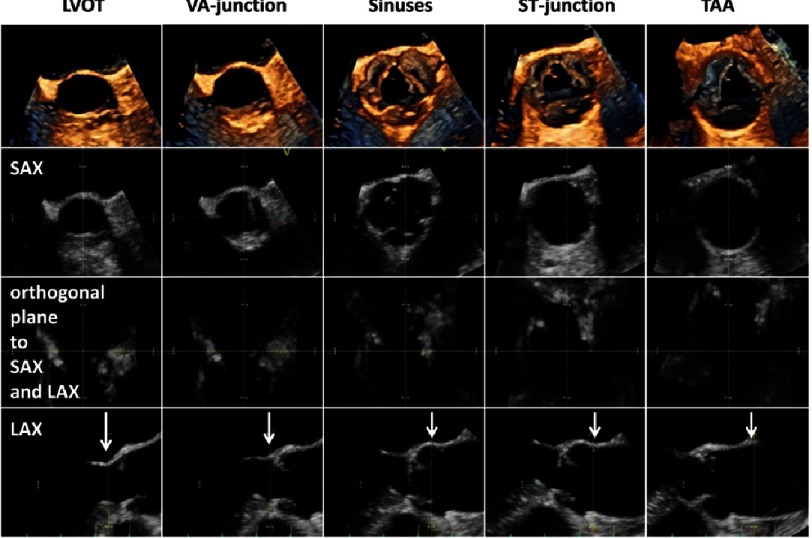
3D data set with correct adjustment of the cardiac structures during mid-systole for accurate measurement of the aortic root diameter. In the first line 3D short axis views against the bloodstream of the LVOT, VA-junction, sinuses of Valsalva, ST-junction and tubular ascending aorta (TAA) are shown. The second line shows corresponding sectional planes of the short axis views in the 3D data set. The third line shows corresponding horizontal views and the fourth line shows corresponding perpendicular long axis views of the aortic root for the correct measurements of cardiac dimensions labeled by white arrows.

A number of the above mentioned challenges and limitations of 2D-echocardiograpghy can be avoided by the use of 3D-echocardiography. Using 3D-echocardiography, the correct sectional planes can be adjusted within a 3D data set at the correct point in the cardiac cycle. Thus, maximum diameters and cross-sectional areas can be objectively controlled and measured by the I-I protocol due to sufficient spatial resolution within the 3D data set during systole (Figure 12). However, the prerequisite for reliable measurements is an acquisition of 3D data sets with optimal image quality using high ultrasound frequencies to get sharper contours of the cardiac structures.

**Figure 13.**
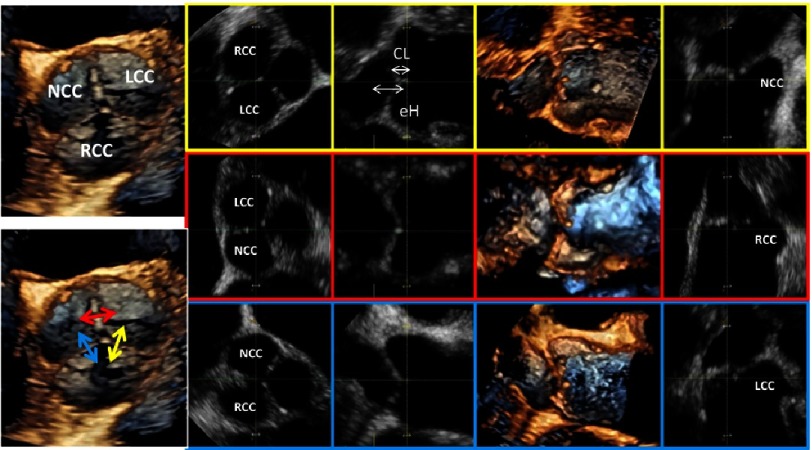
On the left side an en-face view of a normal tricuspid AV is shown. The yellow highlighted images (first line) show the post-processing of correct sectional planes for measurement of CL and eH between the RCC and LCC, the red highlighted images (second line) show corresponding post-processing analysis of CL and eH between the LCC and NCC and the blue highlighted images (third line) show corresponding post-processing analysis of CL and eH between the NCC and RCC.

**Figure 14.**
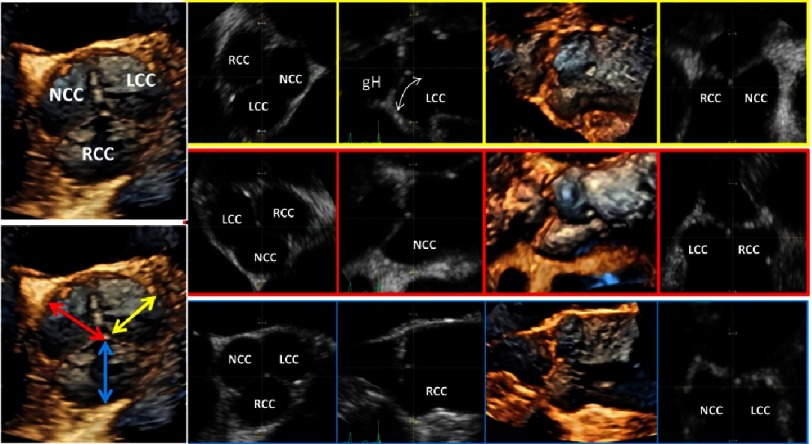
On the left side above an en-face view of a normal tricuspid AV is shown. The yellow highlighted images (first line) show the post-processing of correct sectional planes for measurement of gH of the LCC, the red highlighted images (second line) show corresponding post-processing analysis of gH of the NCC and the blue highlighted images (third line) show corresponding post-processing analysis of gH of the RCC.

3D-echocardiography enables adjustment of the sectional planes through the commissures between the respective cusps near the central point of coaptation for the determination of CL and eH (Figure 13), as well as the sectional planes through the nadirs of each cusp and the center of the free margin for the determination of gH (Figure 14).

**Figure 15.**
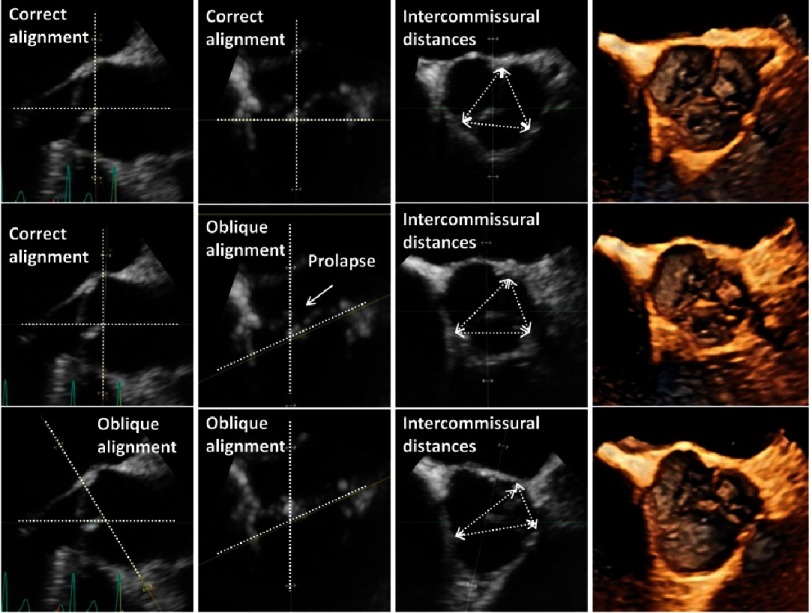
Analysis of the aortic root in correct sectional planes for the determination of the asymmetry by measurements of the intercommissural distances in a patient with prolapse of the NCC. In the first line symmetry of the aortic root is documented by en-face view of the AV displayed by perpendicular planes for adjustment of a correct short axis view parallel to the VA-junction. In the second line an oblique horizontal sectional plane is shown causing significantly different intercommissural distances. In the third oblique horizontal sectional planes and short axis sectional planes are shown causing again misleading measurements.

Symmetric or asymmetric disposition of the aortic root can also be analyzed by adjustments of the correct short axis views (Figure 15). The annulus excursion with respect to rotational and translational movement and the alterations of the angle formed by the mitral and aortic annulus between systole and diastole can be analyzed more reliably using 3D data sets (Figures 10 and 11).

## 3. Assessment of aortic root complex abnormalities

### 3.1. Applying conventional 2D-echocardiography

The systematic echocardiographic assessment of the aortic root complex should focus on the geometry and/or size of the respective cardiac structures which potentially influence valve opening and closure or indicate the risk of aortic rupture^52,67–69,73,74^.

Asymmetry of the aortic root can induce commissural displacement and cusp separation due to cusp restriction, because the free margins of the cusps are directly related to the size of the sinuses of Valsalva. Asymmetry can be described by the size of sinus of Valsalva or by the heterogeneous cusp-to cusp distance. Alternatively, the distance between the commissures can be measured. A difference between the detected distances of >5 mm indicates asymmetry of the sinuses (Figure 15).

Dilatation of the LVOT, basal aortic annulus and aortic root in the presence of normal cusp morphology and motion is described as dilatation of the functional aortic annulus (FAA), which is defined as AR type I corresponding to the Carpentier Classification and causes AR with varying severity.

**Figure 16.**
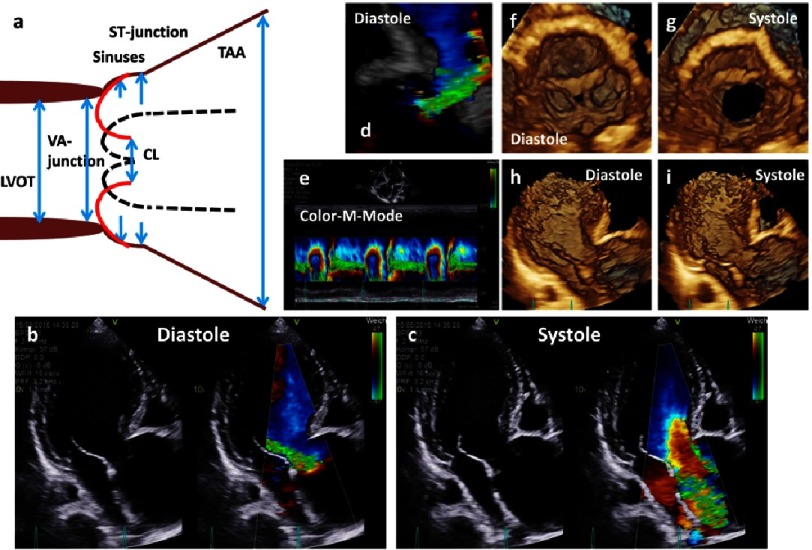
The scheme shows an ectasia of the sinuses of Valsalva and a severe aneurysm of the proximal TAA (a). Below native and color-coded 2D transthoracic images are shown during diastole (b) and systole (c). In addition, 3D TTE image of the aortic regurgitation during diastole (d), color M-Mode of the regurgitation (e), en-face views of the aortic annulus during diastole (f) and systole (g) and 3D transthoracic images of long axis views during diastole (h) and systole (i) are shown.

**Figure 17.**
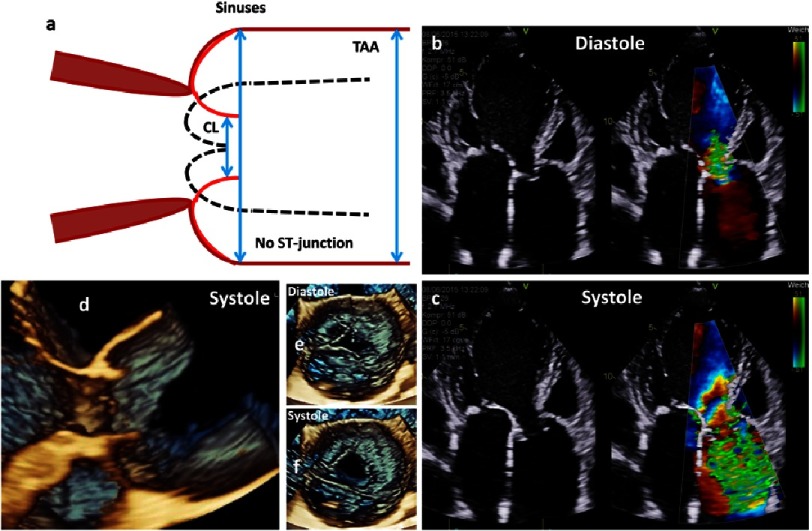
The scheme shows an aneurysm of the sinuses of Valsalva with an ectasia of the proximal ascending aorta and disappearance of the ST-junction and the direct transition of the sinuses into the proximal TAA (a). Further, native and color-coded 2D transthoracic images during diastole (b) and systole (c), 3D transthoracic long axis view during systole (d) and 3D transesophageal en-face views of the AV during diastole (e) and systole (f) are shown.

**Figure 18.**
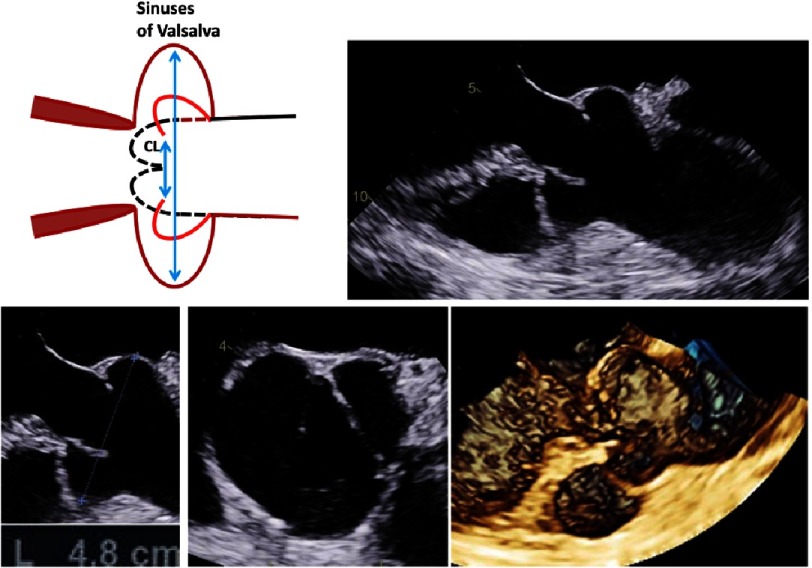
The scheme shows an isolated aneurysm of the sinuses of Valsalva. On the right side and below 2D and 3D transesophageal images during systole as well as the measurement of the aortic root diameter are shown.

**Figure 19.**
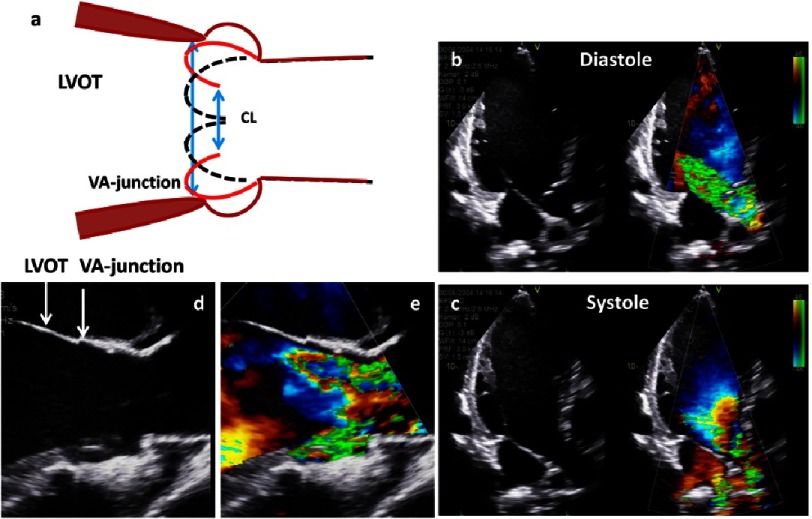
The scheme shows LV dilatation with dilatation of the LVOT and the basal aortic annulus and consecutive severe aortic regurgitation (a). Further, native and color-coded 2D transthoracic images during diastole (b) and systole (c) as well as native (d) and color-coded (e) 2D long axis views of the LVOT and the VA-junction during systole are shown.

**Figure 20.**
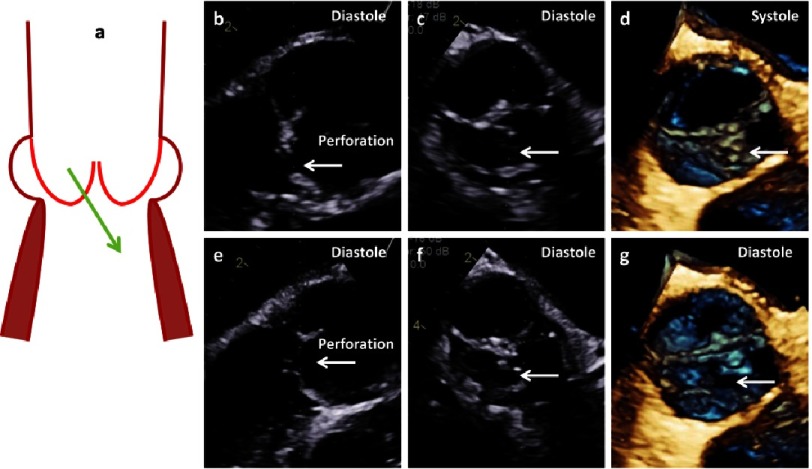
The scheme shows cusp perforation (a). 2D- and 3D images of long axis views (b, e), short axis views (c, f) and 3D-en-face views of the AV (d, g) show perforation of the RCC labeled by white arrows.

**Figure 21.**
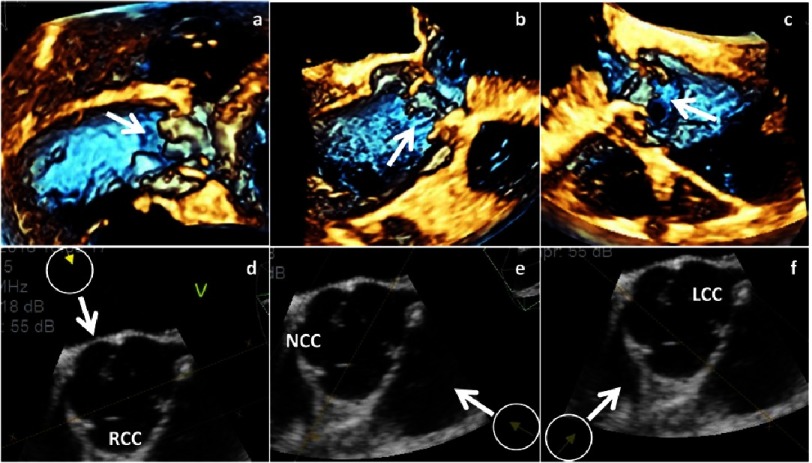
Fenestrations at the margins of the RCC (a), the NCC (b) and the LCC (c) are shown by 3D transthoracic echocardiography. The inhomogeneities of the cusp margin are labeled by white arrows. Below the orientation of the views are given by arrows in corresponding 2D short axis views (d-f).

Type Ia is present if all structures - the basal aortic annulus, aortic root and TAA - are dilated (Figure 16). Type Ib is present, if only the sinuses of Valsalvae and ST-junction are dilated (Figures 17 and 18). Type Ic is characterized by an isolated dilatation of the aortic annulus in relation to the aortic root morphology (Figure 19). Dilatation of the aortic root with normal aortic cusps is documented by a disappearance of ST-junction and a direct transition of the sinuses of Valsalva to the TAA, by a ratio of ST-junction to the aortic annulus >1.5 and by a reduced CL with an increased tenting of the cusp of >11 mm (Figures 16–19). Type Id is characterized by normal morphology of the basal aortic ring and the aortic root in presence of cusp perforation (Figure 20). Cusp perforation has to be distinguished from cusp fenestration, which cannot be visualized by 2D-echocardiography but rather can be documented by en-face views of the cusps during systole using 3d-echocardiography (Figure 21).

### 3.2. Advantages of 3D-echocardiography for the assessment of pathological findings of the aortic root complex

The alignment of the sectional planes at the appropriate position and time within 3D data sets with optimal image quality provides accurate I-I measurements of the maximum dimensions of the respective cardiac structures (Figures 12–15). The en-face view of the sinuses of Valsalva and the commissures of the AV enables an objective documentation of symmetric or asymmetric aortic root (Figure 15).

In general, 3D-echocardiography prevents misleading measurements due to foreshortening views and permits a verification with respect to their correctness. The accurate characterization of the dimensions of the LVOT, basal aortic annulus and aortic root applying 3D-echocardiography facilitates the decision making for appropriate surgical repair technique in these patients.

In AR type Ia ST-junction remodeling by an ascending graft in combination with a potential subcommissural anuloplasty is primary used. In AR type Ib AV sparing procedures, e.g., AV reimplantation or AV remodeling with a subcommissural anuloplasty, in AR type Ic ST-junction anuloplasty and subcommissural anuloplasty, and in AR type Id patch repair techniques of the cusps are generally performed.

## 4. Assessment of aortic valve abnormalities

### 4.1. Applying conventional 2D-echocardiography

**Figure 22.**
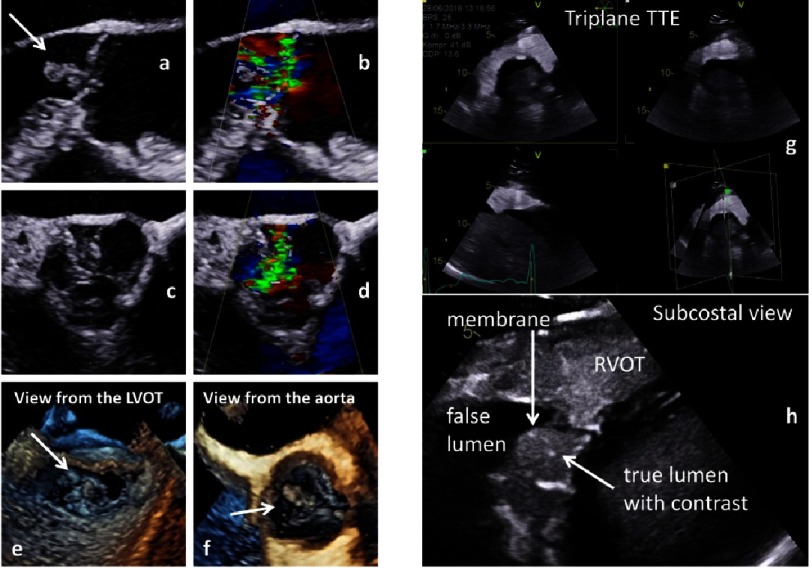
Documentation of vegetations due to endocarditis in native and color-coded 2D transthoracic long axis views (a, b) and 2D transesophageal short axis views (c, d) as well as 3D transesophageal en-face views of the AV from the LVOT (e) and the tubular ascending aorta (f). On the right side aortic dissection (Stanford A) is documented in a triplane subcostal view using contrast echocardiography (g) and in a zoom view of the dissection membrane (h).

Severe pathologies, e.g., aortic dissection and/or endocarditis, have to be detected prior to surgical interventions (Figure 22) because these diseases usually exclude repair strategies^10,16–18^. AV abnomalities due to excessive cusp motion or cusp restriction causes two more AR types^72^.

**Figure 23.**
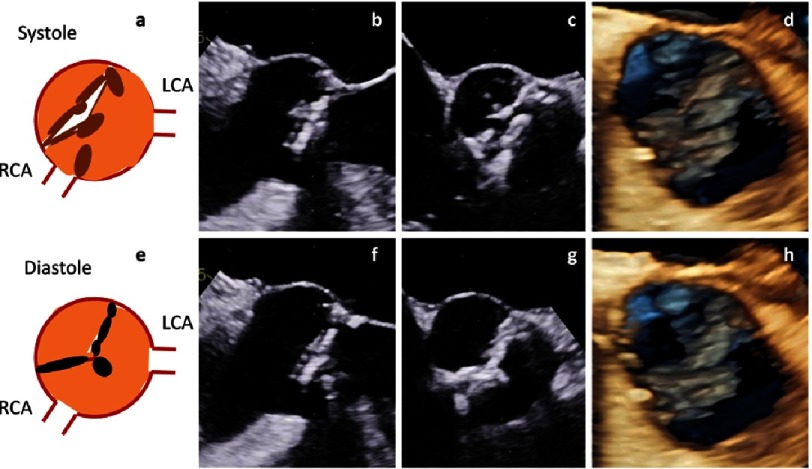
Calcification and retraction of the cusps in a patient with a true bicuspid AV. Scheme of the short axis view (a), 2D transesophageal long axis view (b), 2D transesophageal short axis view (c) and 3D transesophageal en-face view of the AV (d) are shown above during systole. Corresponding views are shown below during diastole (e-h).

**Figure 24.**
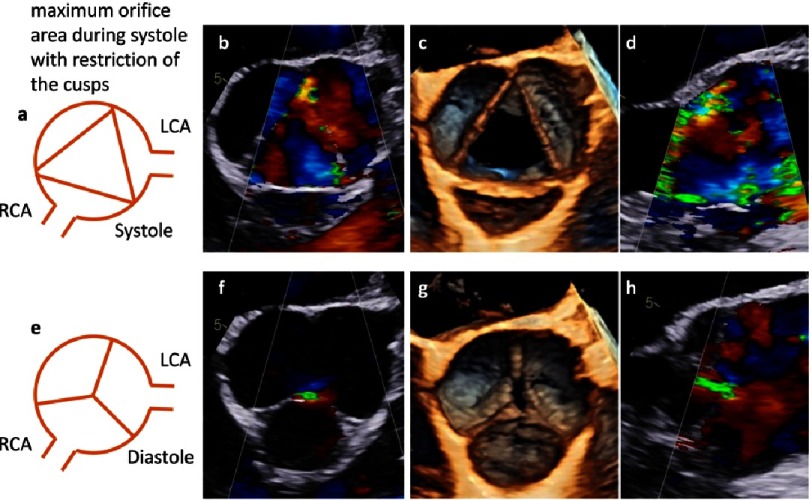
Cusp tethering and functional cusp restriction - tethering is described by reduced geometric cusp height. Scheme of the short axis view (a), color-coded 2D transesophageal short axis view (b), 3D transesophageal en-face view of the AV (c) and color-coded 2D transesophageal long axis view (d) are shown above during systole. Corresponding views are shown below during diastole (e-h).

AR Type II is characterized by cusp prolapse or fenestration of cusps in presence of normal aortic root morphology^67–69,72,75^. AR Type III is characterized by cusp retraction and thickening, often in combination with aortic stenosis due to calcifications (Figure 23). AR can also be induced by functional cusp restriction. Functional restriction is documented by cusp tethering which is caused by dilatation of ST-junction or reduced gHs of the cusps (Figure 24).

The longitudinal view of the AV usually shows sufficiently closed cusps during diastole, in which the nadir of each cusp is the deepest point and where the coaptation is characterized by a CL of 3-5 mm, with a small tenting area or with cusps at the level of the basal aortic annulus without any tenting area. In rare cases billowing and bulging of the cusps without any functional interference can be observed.

**Figure 25.**
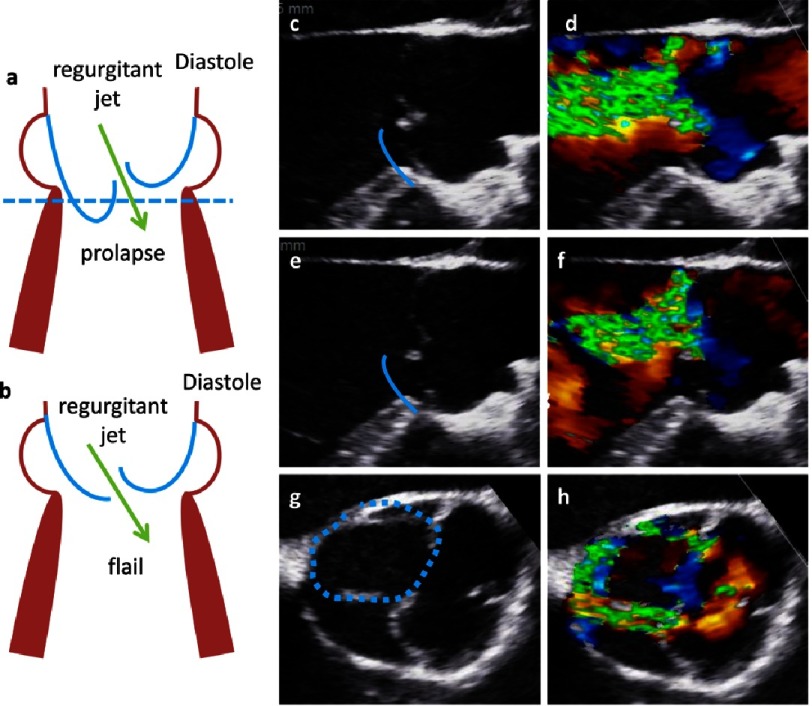
In the scheme a prolapse (a) and a flail (b) are shown in long axis views. On the right side native (c) and color-coded 2D transesophageal long axis view is shown documenting the free margin of the RCC in the LVOT during diastole and the regurgitant jet. Below oblique corresponding views are shown. The course of the RCC and the prolapse are labeled by the blue lines in the native 2D transesophageal images. In (g), (h) color coded 2D transesophageal short axis views show the prolaps by the colored contour displayed by the blue dotted line.

In contrast, in AR type II a prolapse is characterized by a protrusion of a free margin of a cusp into the LVOT resulting in insufficient AV closure, producing AR of varying severity. It is important to distinguish between partial and complete prolapse or flail of one or more cusps. Using 2D-echocardiography a partial prolapse is characterized by a bending of distal parts of one cusp into the LVOT in the long axis view and the documentation of a planar circular structure of the prolapsing cusp in the corresponding short axis view (Figure 25).

A complete prolapse or a flail of one cusp can be characterized by an eversion of the free margin into the LVOT during diastole. Using 2D-echocardiography a large circular or oval annular structure can be documented in presence of a cusp flail in the short axis view. This annular structure is achieved by the fact that parts of the prolapse are below and above the corresponding sectional plane. The eversion of a flail cusp can also be documented in corresponding long axis views through the center of the prolapse (Figure 25).

In AR type III different reasons for stretching or restriction of the free margins of the cusps can be documented (Figure 23). Tethering of normal cusps can be due to dilatation of the aortic root. Stretching and restriction of the cusps are often caused by fibrosis and calcification (Figure 24).

A systematic assessment of the cusps can be summarised by:

1. Describing the number of cusps, which are determined by the number of normal commissures. Unicuspid and bicuspid AV (UAV and BAV) have to be described with respect to the attachment of the commissures, the fusion of cusps with or without raphes and the orientation of the commissure. Another rare AV anomaly is the quadricuspid AV.
2. Characterizing the configuration of the cusps by eH and CL, and the amount of cusp tissue by gH which cannot be accurately determined by 2D-echocardiography. Depending on the topography of the AV, only eH, CL and gH of the RCC can be well estimated by 2D-echocardiography.
3. Assessing additional cusp parameters which are described in the literature but that are often limited and misleading in 2D-echocardiography.
  “Leaflet area” corresponds to the area of each cusp determined in a short axis view.
  “Leaflet height” corresponds to the maximum distance between the central point of the cusp coaptation and the aortic root determined in a short-axis view.
  “Leaflet length” is calculated by the leaflet height and the leaflet depth, which corresponds to the distance between the line from the cusp insertion to the cusp tip and the most convex point of the cusp determined in the long axis view.

All these parameters can be described as surrogate parameters for the characterization of the cusp geometry and morphology. However, cusp morphology seems to be better and more easily assessed by directly measuring CL, eH and gH of each cusp.

### 4.2. Advantages applying 3D-echocardiography

Several aspects for an improved assessment of the AV and its cusps by 3D-echocardiography have to be addressed. The alignment and the orientation of the commissures - especially in UAV and BAV patients - as well as the calcification of the cusps, can be well analyzed and described by en-face views during both systole and diastole. Fenestrations of the cusps can only be visualized during systole, because the margins of the cusps have to be assessed, which is impossible at AV closure during diastole. Thus, fenestrations can only be documented by systolic oblique en-face views of the free-floating cusps (Figure 21).

The main advantage of 3D-echocardiography is that the CL, eH and gH can be determined for each cusp (Figures 13 and 14). The coaptation length should be determined in or near the central point of the AV in corresponding longitudinal sectional planes perpendicular to each coaptation line between two cusps. It is obvious that oblique sectional planes can lead to overestimation of CL and side coil artifacts can interfere with a correct CL measurement in 2D-echocardiography. The coaptation of a tricuspid AV should be described by three CLs of the corresponding cusps. Thus, CL between RCC/NCC, NCC/LCC, and LCC/RCC should be measured in the inner third of the corresponding commissure. Thus, eH should be described in a tricuspid AV by three different eHs for the respective commissure between RCC/NCC, NCC/LCC, and LCC/RCC (Figure 13). The gH has to be determined as a curved length of each cusp during diastole from the nadir of the cusp to the center of the commissure. It is impossible to measure the correct gH by 2D-echocardiography - even of the RCC - because it requires a correct sectional plane orthogonal to the central part of the RCC. Moreover, alignment of the corect sectional planes of the NCC and LCC can only be performed correctly by 3D-echocardiography (Figure 14). In summary, the adequate assessment of cusps morphology by determining CL, eH and gH can only be performed by post-processing in 3D data sets^67–71^.

## 5. Future perspectives

**Figure 26.**
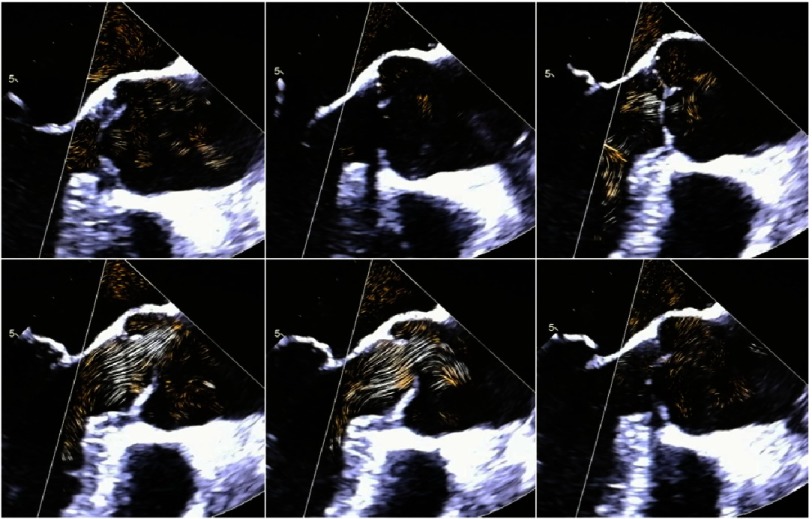
2D transesophageal long axis views within one cardiac cycle showing the blood flow by blood flow speckle tracking. This new technique permits the visualization of the flow vortex.

The preservation of normal dynamics of the basal aortic annulus and the coupling between aortic and mitral annulus can be important factors for long-term survival after AV repair and AV-sparing surgery. Thus, modern echocardiography should focus on rotational and translational motion of the LV and the aortic root. Using 3D-echocardiography, the aortic annulus excursion as well as the angle differences between the mitral and aortic annulus can be objectively analyzed during the cardiac cycle (Figures 10 and 11). In addition, blood flow speckle tracking can be a new approach to characterize pathological flow phenomena in AR patients - especially with eccentric jet formations - in order to get information about potential aortic wall affection due to high shear stress or turbulences (Figure 26).

## Summary

3D-echocardiography should be established in clinical routine to evaluate patients for AV repair and AV-sparing surgery. It is obvious that 3D-transthoracic and transesophageal echocardiography has additional value in characterizing AV and aortic root abnormalities if the image quality of the 3D data sets is sufficient. The assessment of cusp morphology and function can be best performed by CL, eH and gH, which is possible for all cusps using 3D-echocardiography. Moreover, misleading measurements due to non-standardized, oblique sectional planes can be avoided by 3D-echocardiography. Thus, 3D-echocardiography should be mandatory for the analysis of AV and aortic root dimensions as well as the grading of AR severity. However, sufficient training and experience is required before it can be applied in clinical routine.
